# Supplementary material for: Forest carbon sink neutralized by pervasive growth-lifespan trade-offs
Source: Nat Commun. 2020 Sep 8;11:4241. doi: 10.1038/s41467-020-17966-z (PMC7479146; doi:10.1038/s41467-020-17966-z)
Supplement: Supplementary file 1 — Supplementary Information [file 41467_2020_17966_MOESM1_ESM.pdf]

Forest carbon sink neutralized by pervasive growth-lifespan trade-offs

R.J.W. Brienen[1], L. Caldwell[1], L. Duchesne[2], S. Voelker[3], J. Barichivich[4,5], M. Baliva[6], G. Ceccantini[7], A. Di Filippo[6], S. Helama[8], G.M. Locosselli[7], L. Lopez[9], G. Piovesan[6], J. Schöngart[10], R. Villalba[9], E. Gloor[1]*

[1] School of Geography, University of Leeds, Leeds, LS2 9JT, UK
[2] Ministère des Forêts, de la Faune et des Parcs, Direction de la recherche forestière, 2700 Einstein Street, Quebec City, Quebec, G1P 3W8, Canada.
[3] Dept of Environmental and Forest Biology, SUNY-ESF, Syracuse, New York 13210, USA
[4] Laboratoire des Sciences du Climat et de l'Environnement, IPSL, CNRS/CEA/UVSQ, 91191 Gif sur Yvette, France.
[5] Instituto de Geografía, Pontificia Universidad Católica de Valparaíso, Valparaíso, Chile
[6] Department of Agriculture and Forest Sciences (DAFNE), University of Tuscia, Via SC de Lellis, 01100, Viterbo Italy.
[7] University of São Paulo, Institute of Biosciences, Department of Botany, Rua do Matão, 277, 05508-090, São Paulo, SP, Brazil.
[8] Natural Resources Institute Finland, Ounasjoentie 6, 96200 Rovaniemi, Finland
[9] Instituto Argentino de Nivología, Glaciología y Ciencias Ambientales (IANIGLA), CONICET-Mendoza, C.C. 330, (5500) Mendoza, Argentina
[10] Instituto Nacional de Pesquisas Da Amazônia (INPA), Coordenação de Dinâmica Ambiental (CODAM), Av. André Araújo 2936, 69067-375 Manaus, Brazil.

*Corresponding Author: Roel Brienen ; r.brienen@leeds.ac.uk

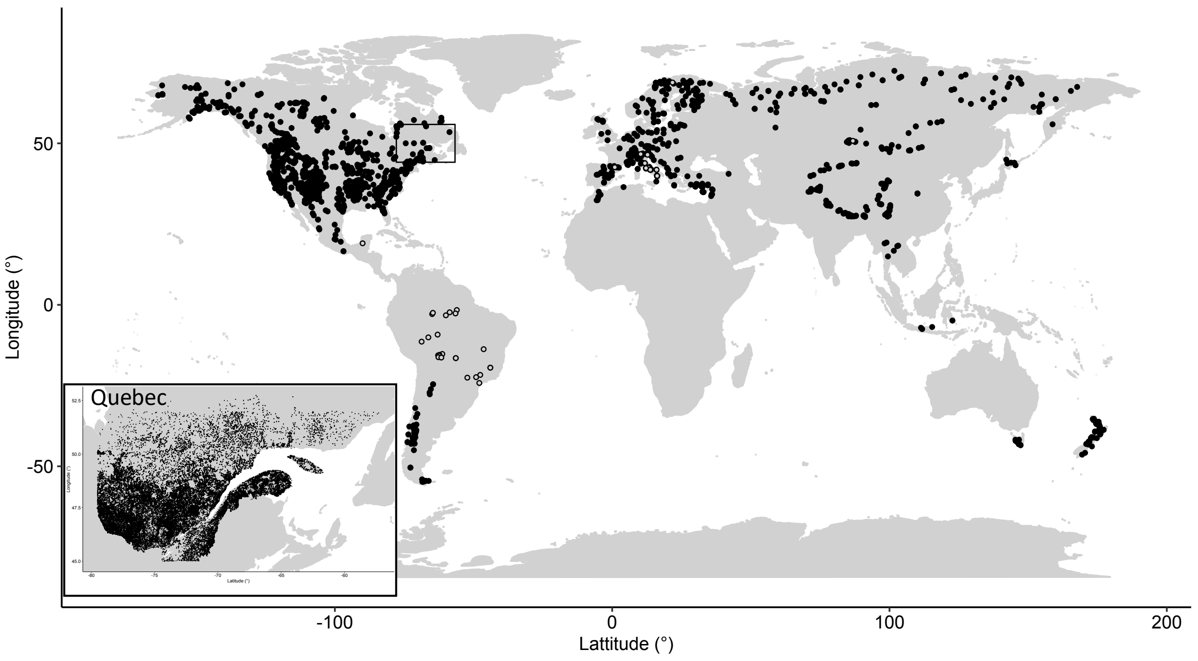

Supplementary Figure 1. Sampling locations for all sites included in this study. Closed symbols indicate the International Tree Ring Data Bank (ITRDB [1]) sites, open symbols addional published and unpublished data that were used here. Inset shows the sampling locations from Quebec from Ministère des Forêts de la Faune et des Parcs[2]. Overview of the different datasets used is provided in Supplementary Table 1. All site coordinates are provided in accompanying metadata (see "Data availability" statement in main article).

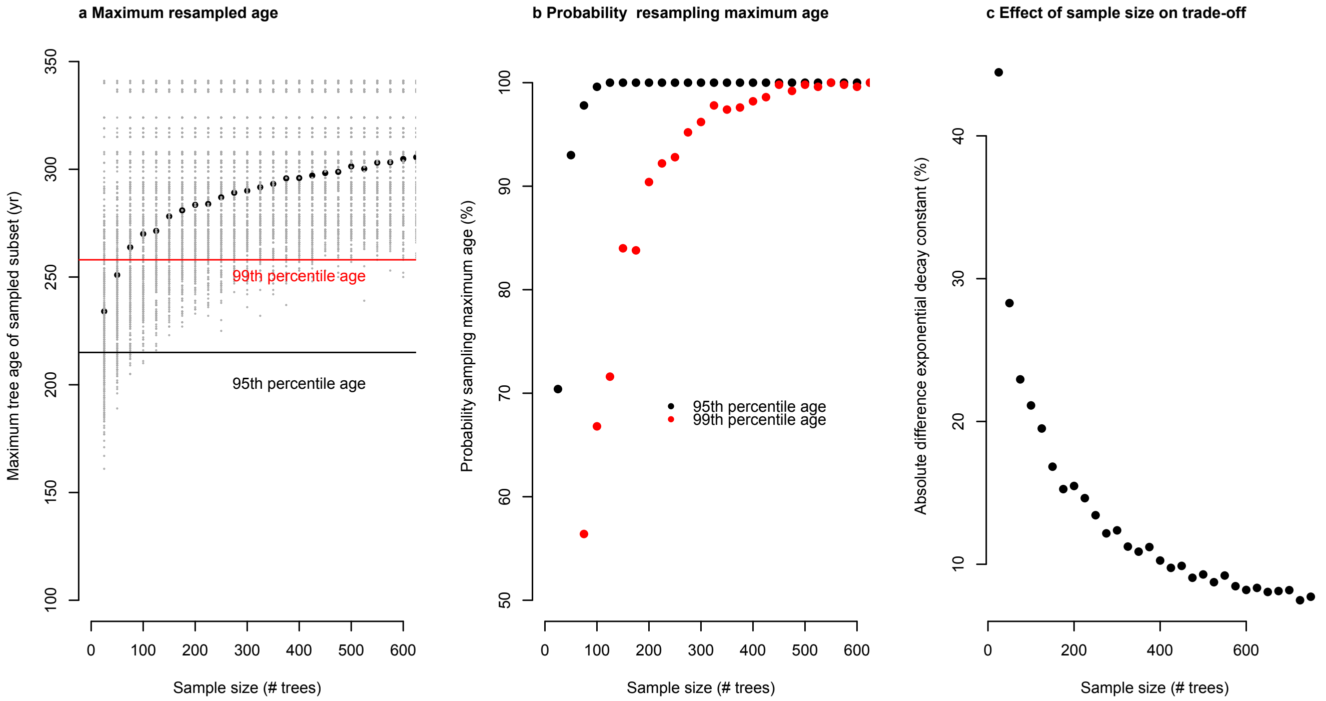

Supplementary Figure 2a-c. Effect of sample size on probability of capturing maximum tree age (a,b) and on accuracy to estimate the trade-off strength (c). Relationship between sample size and maximum tree age (a). This relationship was established by randomly resampling tree ring data from 11.752 *Picea mariana* trees from Quebec-NFI data from sites north of 50.7°N. We varied the sample size from 25 to 600 trees, and repeated this resampling procedure 500 times. Points in panel a indicate the maximum ages for each random subset (grey points) and the mean of the maximum ages (black points). Lines in panel a indicate the 95th and 99th percentile age across all 11.752 *Picea mariana* trees. Panel b shows the probability of capturing the 95th and 99th percentile ages (black, red) for each sample size. This shows that for example a sample of 100 trees captures in 65% of the cases the 99th percentile age and captures 100% of the 95th percentile age. Panel c shows the effect of sample size on the accuracy of the estimation of the trade-off for full *Picea mariana* dataset using the negative exponential decay constant.

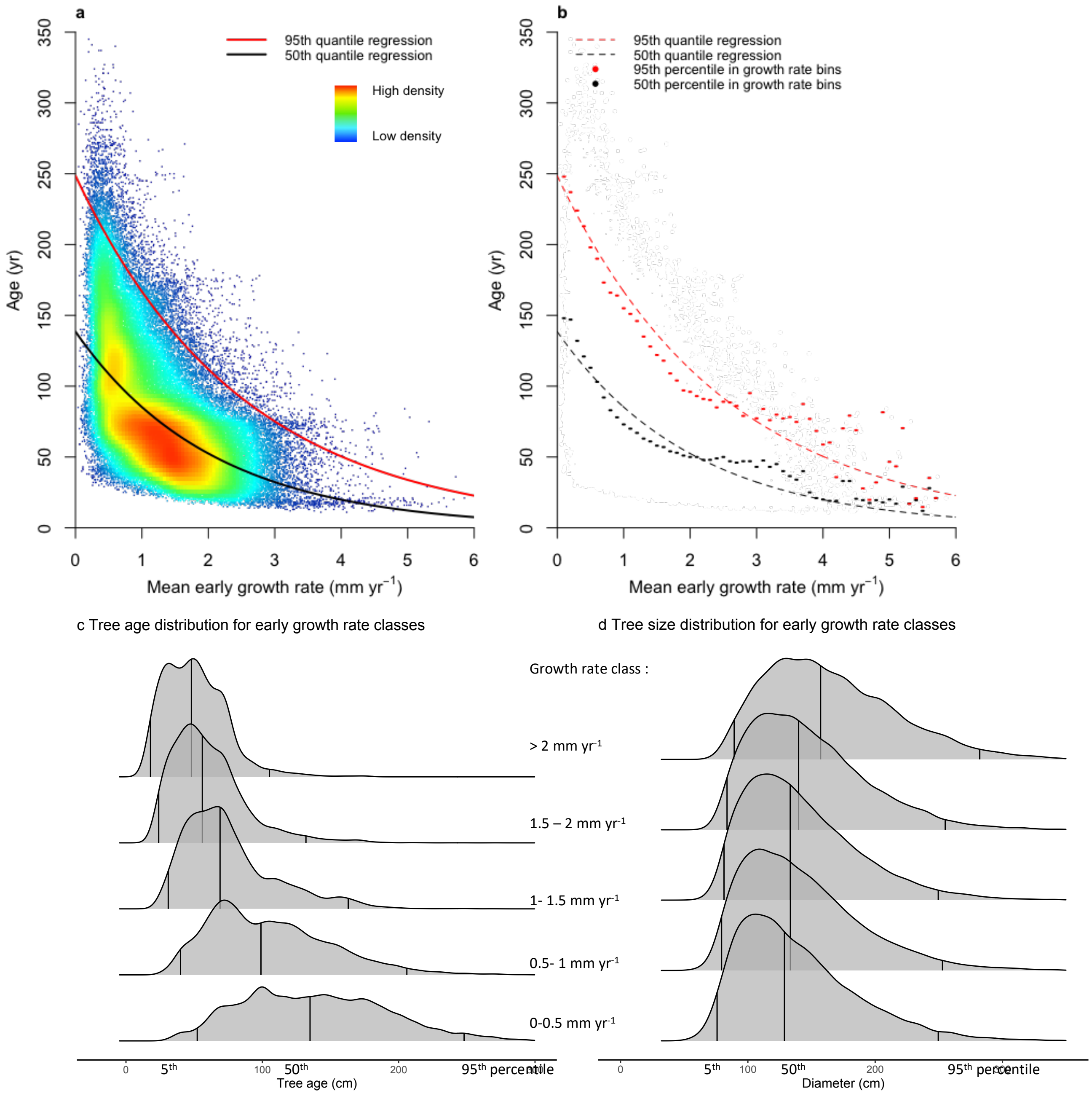

Supplementary Figure 3a-d. Relationship between mean early growth rate and age and diameter for *Picea mariana* (n= 77.691 trees). a, Density plot of early growth rate versus age with trend estimates using quantile regressions of a negative exponential form on the 50th, or median (black line), and 95th percentiles (red line). **b,** Estimation of the 50th and 95th percentile ages in mean early growth rate bins (of 2 mm widths). Lines of quantile regression from panel a are plotted for comparison purposes. **c,d.** Tree age and tree size distributions for five early growth rate classes for *Picea mariana*, Quebec, Canada. Early growth rates were calculated for the first 10 years of trees life. Lines indicate the lowest 5th, 50th and 95th percentile ages.

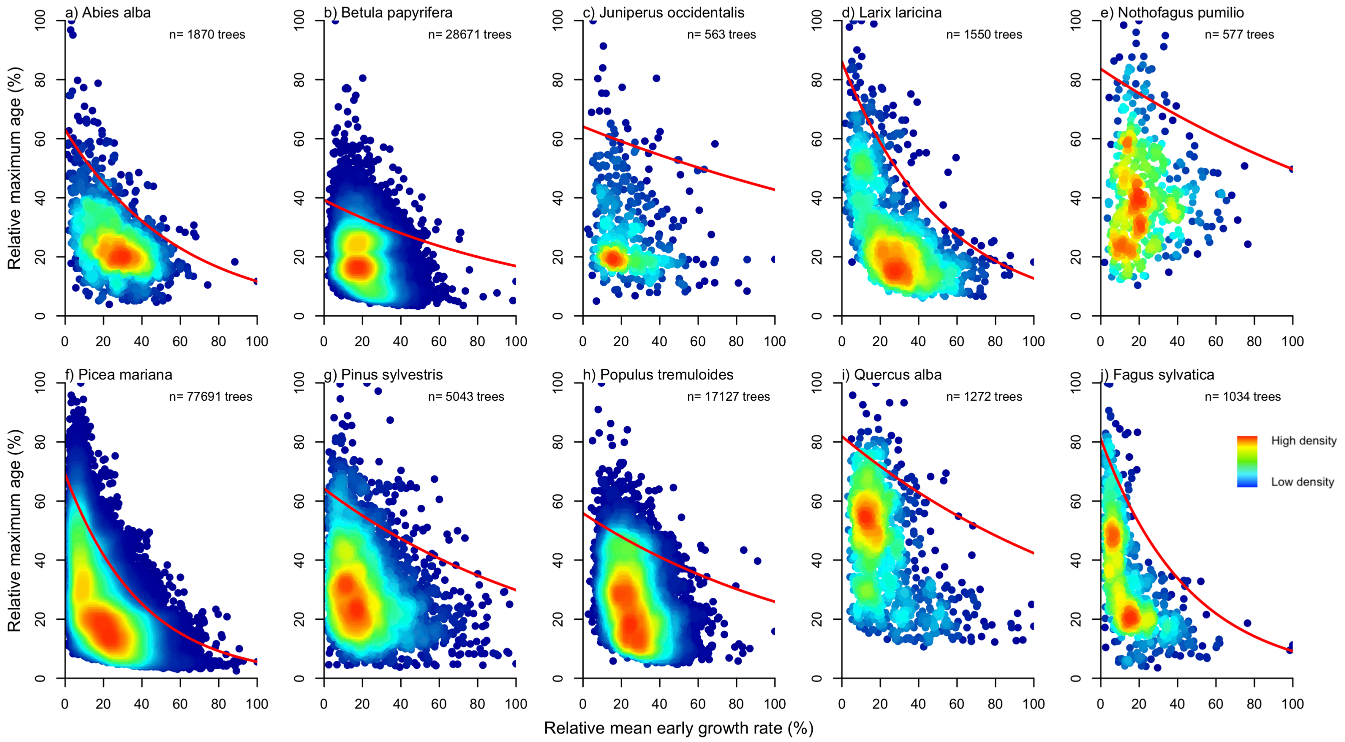

Supplementary Figure 4a-j, Relationship between mean early growth rate and age for ten species shown in Figure 1c. Relative mean early growth rate and relative lifespan were calculated as the ratio (in %) of the early growth rate or age of each tree relative to the maximum early growth rate or maximum age for each species.

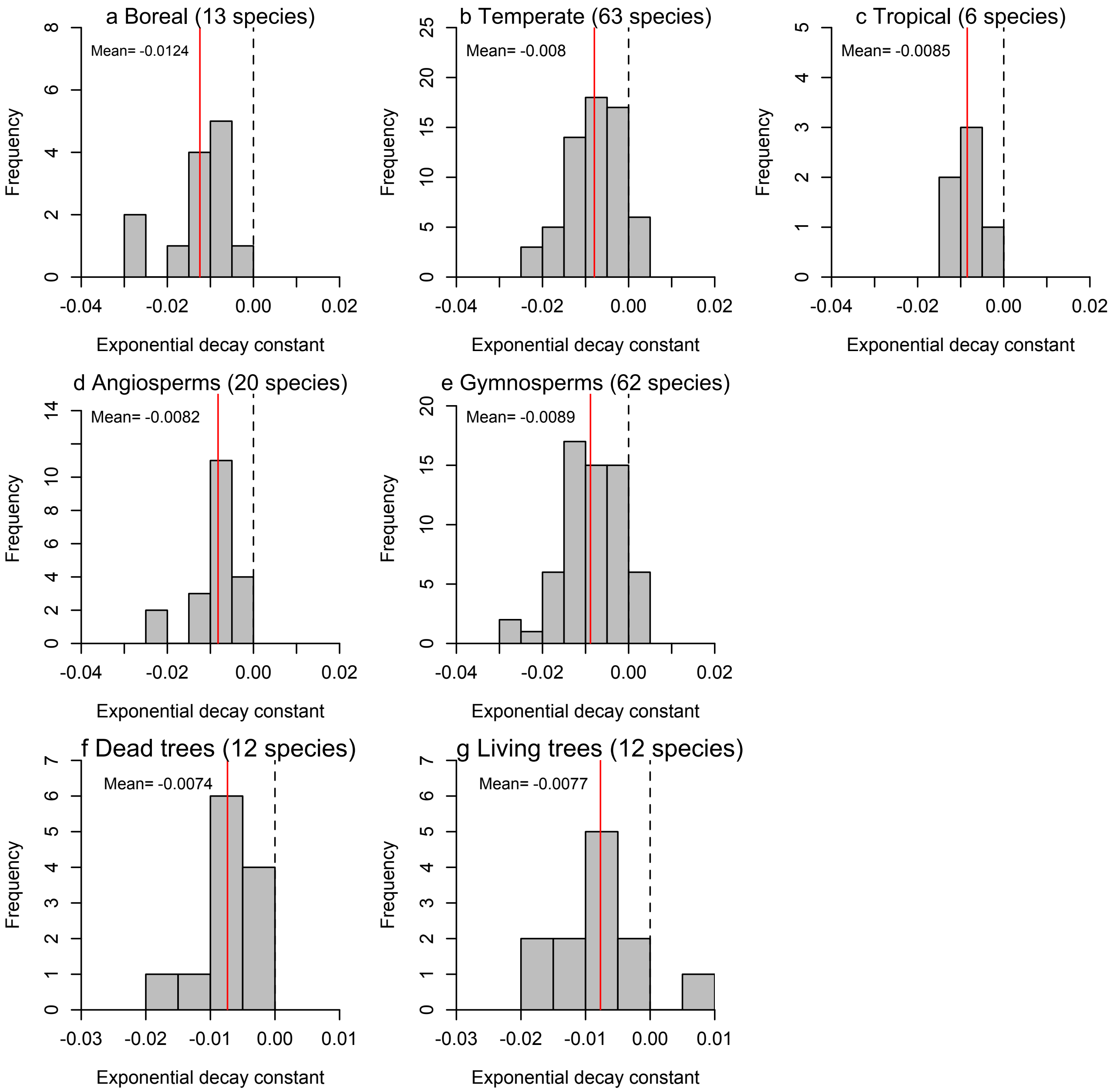

Supplementary Figure 5 a-g. Histogram of the exponential decay constant of relative early growth rate vs. relative lifespan relationships for 82 species separated into their main growing climate (a-c), major plant taxa (d,e), and into living and dead trees (f,g). Species were assigned to a climate zone using their distribution from our own dataset, with boreal species defined as species occurring north of 60ºN, and tropical species as those confined to 20ºN-20ºS. For comparison of dead and living trees, we used 12 species that had sufficient data-availability for both categories (> 150 trees). There is no significant difference in slopes between dead and living trees (two-sided paired t.test, t= -0.1095, p= 0.915, n = 12).

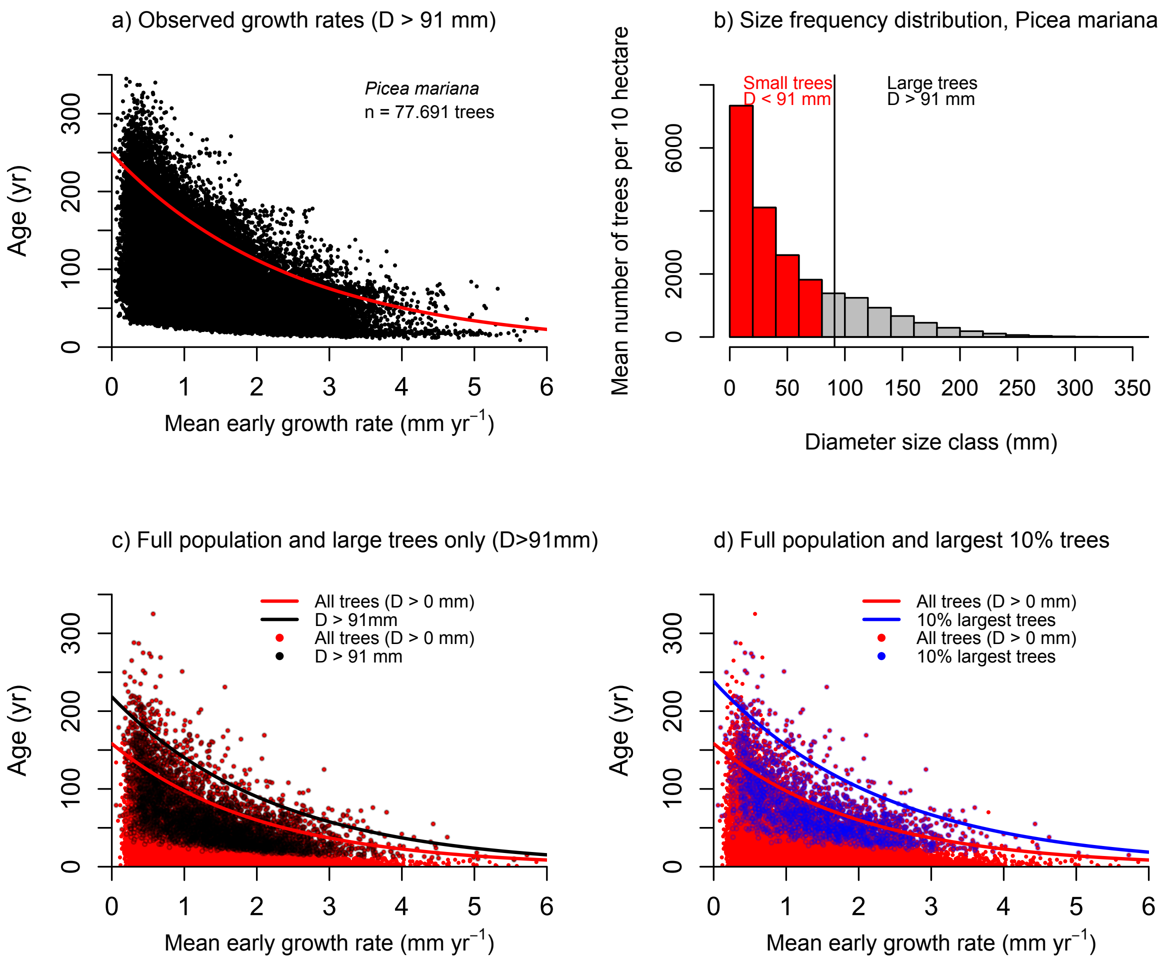

Supplementary Figure 6a-c. Assessment of the effect of the large tree selection bias[3] on the strength of the trade-off between early growth and tree lifespan. a, Observed *Picea mariana* growth rates along with an estimation of the trade-off using the 95th quantile regression. Note here the missing tree ring width data for trees smaller than 91 mm (i.e., empty space in the lower left corner of the graph). **b,** Mean population size distribution of *Picea mariana* derived from the Quebec NFI plot data, with red bars indicating size classes that were not sampled in the Quebec NFI tree ring dataset (i.e, trees with diameter < 91 mm). **c,** Comparison of early growth versus age relationships for the original tree ring dataset, excluding trees < 91 mm in diameter (black points), and for a realistic reconstruction of the full population, including all trees > 0 mm in diameter (red points). These reconstructions were obtained by random resampling from original tree ring data (see methods). d, Comparison of an alternative sampling scenario where all 10% largest trees are sampled (blue points) versus all trees in population (red points). Regression lines in panels a, c and d are 95th quantile regressions using negative exponential function between early growth and tree age.

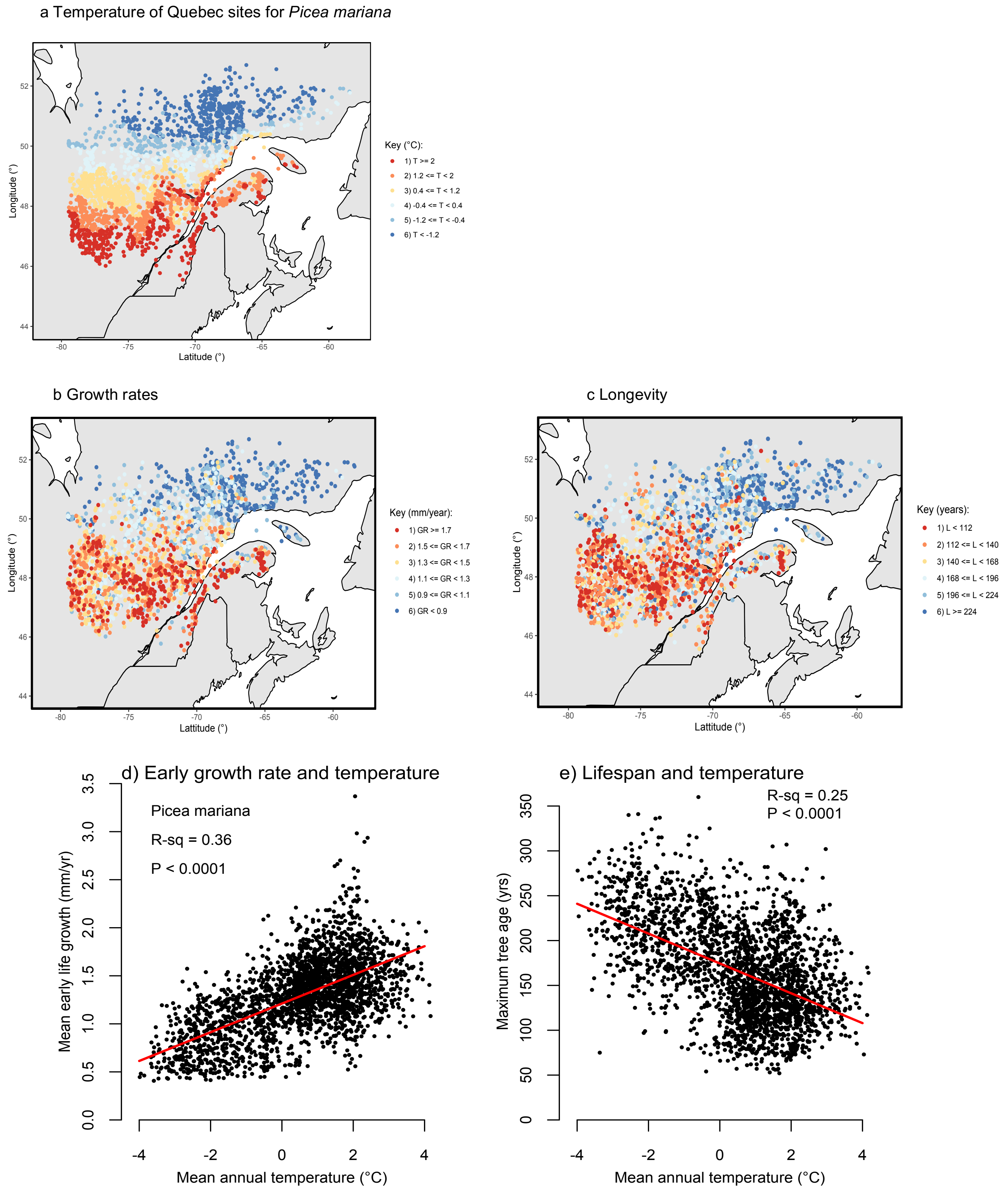

Supplementary Figure 7a-e. Spatial variation in temperature, early growth, maximum age, and early growth and lifespan relationships with temperature for *Picea mariana* in Quebec. Linear regressions were used to assess relationships between mean annual temperature and early growth and lifespan (d and e). Early growth rates were calculated for the first 10 years of trees life.

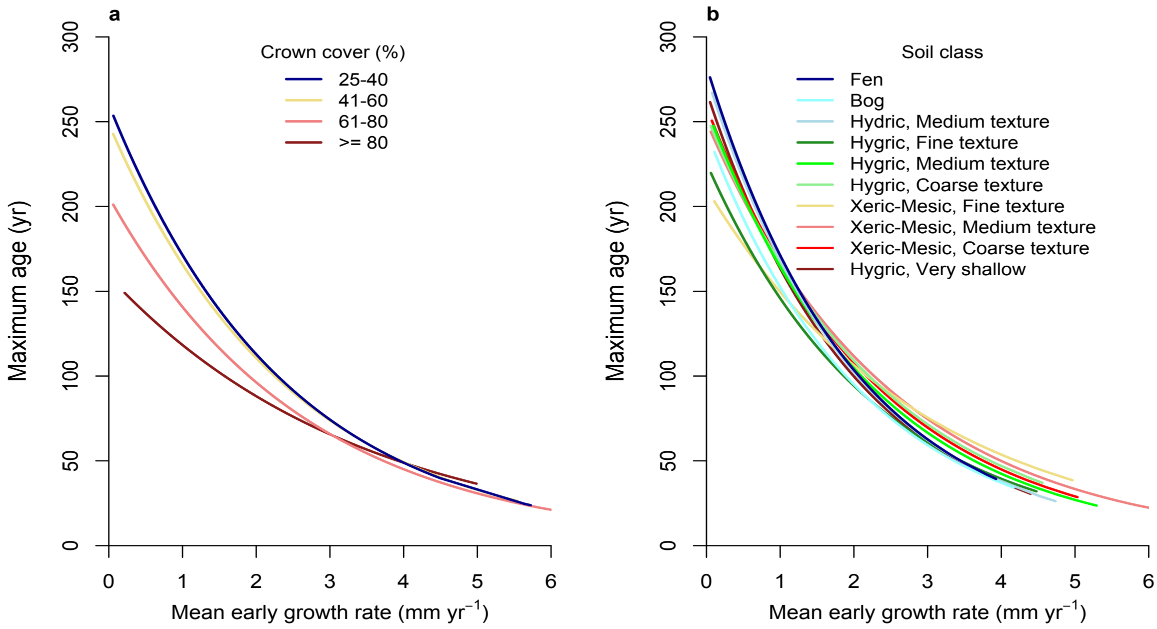

Supplementary Figure 8a,b. Relationship between mean early growth rate and maximum lifespan for *Picea mariana* trees from Quebec within fixed crown cover classes (a) and within fixed soil types (b). The early growth rate -lifespan relationship were estimated using negative exponential 95th quantile regressions. Crown cover and soil type information comes from Quebec-National Forestry Inventory data. Detailed descriptions of soil physical environment classes is given in reference 4. Early growth rates were calculated for the first 10 years of trees life.

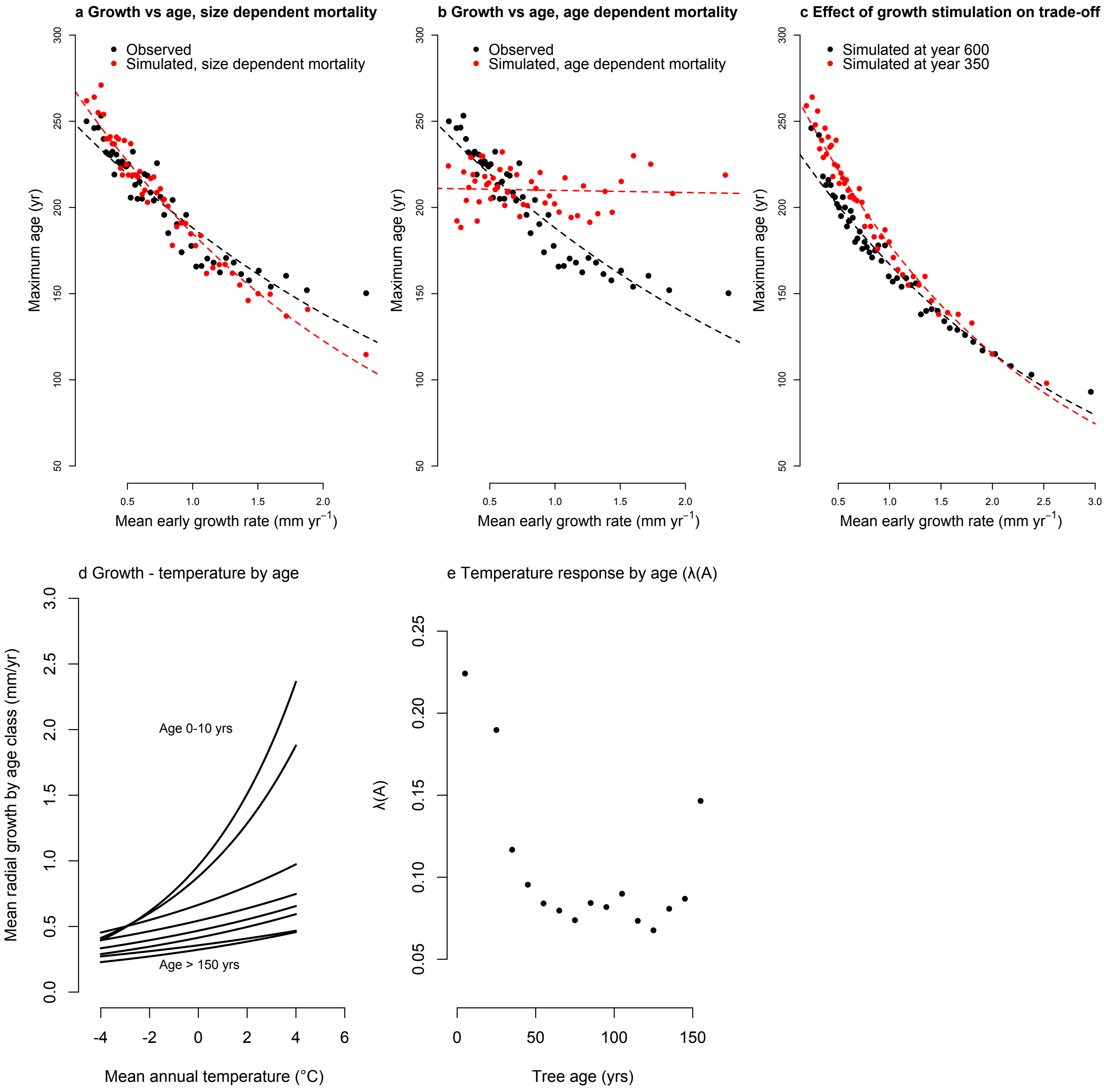

Figure 9a-d. Comparison of observed and simulated trade-offs, and growth response of *Picea mariana* to temperature. a, Observed trade-offs between early growth rate and maximum tree age are for *Picea mariana* from sites north of 50.7°N. Simulated trade-offs for the size dependent mortality formulation are for trees that were alive at year 600 and larger than 91 mm in diameter. **b,** Simulated trade-offs for the age dependent mortality formulation are for trees alive at year 600 in the growth simulation and older than 74 years. **c,** The effect of recent growth increases on the trade-off between early growth and tree age by sampling from the simulated tree ring trajectories at the end of the growth increase period, at year 350, and at year 600, without any recent growth increases (see main ms Fig. 3e). Points in panels a-c represent the oldest 95th percentile ages in early growth rate bins of variable widths and equal number of trees in each bin. Early growth rate-lifespan relationship were estimated using negative exponential 95th quantile regressions. **d,** Growth response to temperature calculated in eight different age bands of 10 years (0-10, 10-20, …. 140-150, >150 yr) for the full *Picea mariana* dataset from Quebec using an exponential form and major axis regression to estimate the relationship between temperature and growth. **e,** Growth response to temperature for different size classes as a function of tree age.

Supplementary Table 1 Data sources, locations and sample size for tree ring data used in this study

Data	Locations	#Sites	# Species	# Trees	Observations	References
National Forestry Inventory- Quebec	Quebec, Canada	68.316	12	158.318	Living trees only	MFFP[2]
International Tree-Ring Data Bank (ITRDB)	Global	1997	99	51.182	Dead and living trees	ITRDB[1]
Cedrela odorata	Mexico & Bolivia	3	*1*	279	Living trees only	Brienen et al. (2010)[5]
Hymenaea spp	Brazil	12	*2*	204	Living trees only	Locosselli et al.(2017) [6]
Macrolobium acaciifolium	Brazil	4	*1*	101	Living trees only	Schöngart et al. (2005) [7]
Centerolobium microchaete	Bolivia	7	1	131	Living trees only	Lopez et al. (2005) [8]
Fagus sylvatica	Italy	12	1	731	Living trees only	Di Filippo et al. (2012) [9]
Pinus heldreichii	Italy	15	*1*	117	Living trees only	Piovesan et al. (2019) [10]
Pinus montana	Switzerland	1	*1*	191	Dead and living trees	Bigler (2016) [11]
Pinus sylvestris	Finland	various	*1*	1200	All subfossil trees	Helama et al. (2004) [12]
Pinus uncinata, Larix sibirica	Pyreness &Russian Altai	various	*2*	1763	Dead and living trees	Büntgen et al. (2019) [13]
Pinus echinata, Quercus stellata	Ozarks, Missouri, USA	various	2	296	Data for *Quercus stellata* unpublished.	Voelker et al. (2006) [14]

	Overall analysis	Within species trade- offs
Number of species	110	82
Number of tree records	214.330	211.165
Number of sites	70.383	70.257

References.

1 https://www.ncdc.noaa.gov/data-access/paleoclimatology-data/datasets/tree-ring

2 MFFP. Ministère des Forêts de la Faune et des Parcs. Norme d'inventaire écoforestier, Placettes-échantillons temporaires, Édition 2016. Available: https://www.mffp.gouv.qc.ca/publications/forets/connaissances/Norme-PET.pdf (2016).

3 Brienen, R. J. W., Gloor, E. & Zuidema, P. A. Detecting evidence for CO_2 fertilization from tree ring studies: The potential role of sampling biases. *Global Biogeochemical Cycles* **26**, GB1025 (2012).

4 Duchesne, L., Houle, D., Ouimet, R., Lambert, M.-C. & Logan, T. Aboveground carbon in Quebec forests: stock quantification at the provincial scale and assessment of temperature, precipitation and edaphic properties effects on the potential stand-level stocking. *PeerJ* **4**, e1767-e1767 (2016).

5 Brienen, R. J. W., Zuidema, P. A. & Martinez-Ramos, M. M. Attaining the canopy in dry and moist tropical forests: strong differences in tree growth trajectories reflect variation in growing conditions. *Oecologia* **163**, 485-496 (2010).

6 Locosselli, G. M., Krottenthaler, S., Pitsch, P., Anhuf, D. & Ceccantini, G. Age and growth rate of congeneric tree species (*Hymenaea* spp.– Leguminosae) Inhabiting different tropical biomes. *Erdkunde* **71**, 45-57 (2017).

7 Schöngart, J., Piedade, M. T. F., Wittmann, F., Junk, W. J. & Worbes, M. Wood growth patterns of *Macrolobium acaciifolium* (Benth.) Benth. (Fabaceae) in Amazonian black-water and white-water floodplain forests. *Oecologia* **145**, 454-461, doi:10.1007/s00442-005-0147-8 (2005).

8 López, L., Rodríguez-Catón, M. & Villalba, R. Convergence in growth responses of tropical trees to climate driven by water stress. *Ecography* (2019).

9 Di Filippo, A., Biondi, F., Maugeri, M., Schirone, B. & Piovesan, G. Bioclimate and growth history affect beech lifespan in the Italian Alps and Apennines. *Global Change Biol.* **18**, 960-972, doi:10.1111/j.1365-2486.2011.02617.x (2012).

10 Piovesan, G. *et al.* Tree growth patterns associated with extreme longevity: Implications for the ecology and conservation of primeval trees in Mediterranean mountains. *Anthropocene* **26**, 100199 (2019).

11 Bigler, C. Trade-offs between growth rate, tree size and lifespan of mountain pine (*Pinus montana*) in the Swiss National Park. *PloS one* **11**, e0150402 (2016).

12 Helama, S., Lindholm, M., Timonen, M. & Eronen, M. Dendrochronologically dated changes in the limit of pine in northernmost Finland during the past 7.5 millennia. *Boreas* **33**, 250-259 (2004).

13 Büntgen, U. *et al.* Limited capacity of tree growth to mitigate the global greenhouse effect under predicted warming. *Nature communications* **10**, 2171 (2019).

14 Voelker, S. L., Muzika, R. M., Guyette, R. P. & Stambaugh, M. C. Historical CO_2 growth enhancement declines with age in *Quercus* and *Pinus*. *Ecol. Monogr.* **76**, 549-564 (2006).
